# Relapsing fever in young refugees from East Africa

**DOI:** 10.1186/s13054-017-1777-z

**Published:** 2017-08-03

**Authors:** Spinello Antinori, Valeria Colombo, Mario Corbellino

**Affiliations:** 1 Department of Biomedical and Clinical Sciences “Luigi Sacco”, Università degli Studi di Milano, Via GB Grassi, 74, 20157 Milano, Italy; 2 III Division of Infectious Diseases, ASST Fatebenefratelli Sacco, Milano, Italy

We read with interest the letter by Cutuli et al. [1] describing a case of severe co-infection by *Leptospira* spp. and *Borrelia recurrentis* in a young female refugee from East Africa. We would like to comment on several issues raised by their paper.

First, the authors state “nits were present on her scalp…”. In our opinion, this sentence may mislead readers as meaning that “head lice” (*Pediculus humanus capitis*) are the vectors of louse-borne relapsing fever (LBRF). Indeed, body lice (*Pediculus humanus humanus*) are to date the only demonstrated vectors of the disease. We are aware of only two reports providing proof that head lice can harbor *B. recurrentis* and consequently might act as a vector of LBRF [2, 3]. However, in the study conducted in Ethiopia, body lice had concomitantly infested the patients and thus the authors could not formally exclude that the latter were not implicated as vectors of the disease [2].

The second point that needs clarification is the relative contribution of the two microorganisms (i.e*.*, *Leptospira* and *Borrelia*) to the described clinical picture: shock, acute respiratory distress syndrome (ARDS), renal failure, jaundice, anemia, thrombocytopenia, altered blood coagulation, and high procalcitonin. With the exclusion of ARDS, which may correctly be attributed to severe leptospirosis, all other findings can be observed in patients with severe clinical presentation of both diseases. However, in the recent wave of LBRF observed in Europe, intensivists were faced with severe cases of LBRF presenting with shock solely as a consequence of the Jarisch-Herxheimer reaction precipitated by administration of antibiotics [4].

A final point that deserves comment concerns the microbiology diagnosis. Molecular biology by means of real-time polymerase chain reaction in this case provided the correct diagnosis of both infections. Although not clearly stated, the authors report that malaria was excluded among other possible differential diagnoses. Thus, we can infer that blood smears examined were negative not only for *Plasmodium* species but also for *Borrelia*. This fact may hint at the possibility that the patient suffered from a relapse and not a first episode of LBRF although Cutuli et al. state that “she had no relevant past medical history”. Given the linguistic barrier encountered with refugees, a previous episode might have been overlooked. Finally, although LBRF was not mentioned in a review dealing with diagnosis and management of critically ill migrants [5], intensivists should be aware of the increasing frequency of this infectious disease.

## Abbreviations

ARDS: Acute respiratory distress syndrome
LBRF: louse-borne relapsing fever
