# Supplementary material for: Relapsing fever in young refugees from East Africa
Source: Crit Care. 2017 Aug 3;21:205. doi: 10.1186/s13054-017-1777-z (PMC5543426; doi:10.1186/s13054-017-1777-z)
Supplement: Related texts

# Reply: Authors’ response

**Authors:** Salvatore L. Cutuli, Gennaro De Pascale, Alessandra Ciervo, Massimo Antonelli

We are pleased to answer the issues raised by Antinori and coworkers over the case of a young critically ill female refugee who developed ARDS and multi-organ dysfunction (MOD) associated with *B. recurrentis* and *Leptospira* species co-infection [6].

First, we confirm that the patient had plenty of body lice but the nits were first identified on her scalp. Second, we cannot identify the specific role of the single agent (*Borrelia* or *Leptospira*) in the severe clinical picture, although this was not meaningful from a clinical point of view. Lastly, we cannot exclude that the patient suffered from a relapse rather than a first episode of *Borrelia* infection, and the information we retrieved from her relatives (who could speak French), were not useful for clarifying this issue.

We are grateful to Antinori and coworkers for their interesting feedback. We think that our experience witnesses the tricky role of medical staff dealing with a life-threatening clinical picture, re-emerging diseases, and difficult health care organization.

Collaboration among infectious disease specialists, microbiologists, and ICU physicians may be key to solving this issue.
